# The association of PTPN22 rs2476601 polymorphism and CTLA-4 rs231775 polymorphism with LADA risks: a systematic review and meta-analysis

**DOI:** 10.1007/s00592-014-0613-z

**Published:** 2014-07-09

**Authors:** Fang Dong, Guang Yang, Hong-Wei Pan, Wei-Huang Huang, Li-Peng Jing, Wen-Kai Liang, Na Zhang, Bao-Huan Zhang, Man Wang, Yang Liu, Li-Ju Zhang, Si-Heng Zhang, He Li, Chuan Chen, Li-Hong Nie, Chun-Xia Jing

**Affiliations:** 1 Department of Epidemiology, Medical School, Jinan University, Guangzhou, 510632 Guangdong China; 2 Department of Parasitology, Medical School, Jinan University, Guangzhou, 510632 Guangdong China; 3 Department of Ophthalmology, Medical School, Jinan University, Guangzhou, Guangdong China; 4 Department of Endocrine, The First Affiliated Hospital, Jinan University, Guangzhou, Guangdong China

**Keywords:** PTPN22, CTLA-4, Polymorphism, LADA, Systematic review, Meta-analysis

## Abstract

Although the polymorphisms of PTPN22 and the variants of CTLA-4 have been reported to be the susceptibility genes, which increased risk of latent autoimmune diabetes in adults (LADA), the results remained inconclusive. The aim of this meta-analysis was to evaluate the association between the polymorphisms of two genes and LADA. We performed a systematic review by identifying relevant studies and applied meta-analysis to pool gene effects. Data from ten studies published between 2001 and 2013 were pooled for two polymorphisms: rs2476601 in the PTPN22 gene and rs231775 in the CTLA-4 gene. Data extraction and assessments for risk of bias were independently performed by two reviewers. Fixed-effect model and random-effect model were used to pool the odds ratios; meanwhile, heterogeneity test, publication bias and sensitive analysis were explored. The minor T allele at rs2476601 and the minor G at rs231775 carried estimated relative risks (odds ratio) of 1.52 (95 % CI 1.29–1.79) and 1.39 (95 % CI 1.11–1.74), respectively. These alleles contributed to an absolute lowering of the risk of all LADA by 4.88 and 14.93 % when individuals do not carry these alleles. The estimated lambdas were 0.49 and 0.63, suggesting a codominant model of effects was most likely for two genes. In summary, our systematic review has demonstrated that PTPN22 rs2476601 and CTLA-4 rs231775 are potential risk factors for LADA. An updated meta-analysis is required when more studies are published to increase the power of these polymorphisms and LADA.

**Electronic supplementary material:**

The online version of this article (doi:10.1007/s00592-014-0613-z) contains supplementary material, which is available to authorized users.

## Introduction

Latent autoimmune diabetes in adults (LADA) is commonly considered as a type of autoimmune diabetes that resembles type 1 diabetes (T1D); however, it masqueraded as type 2 diabetes (T2D) in the initial stage [1–3]. It is commonly recognized that LADA as the subgroup of adult phenotypic type 2 diabetes patients is positive for a GAD antibody [4]. Because of its clinical manifestation exhibits both presentation of two type diabetes, alternative terms have been used to describe this condition as type 1.5 diabetes [5]. There are 347 million people worldwide have diabetes, and LADA accounts for 2–12 % of all cases of diabetes [6].

The patients with LADA were present autoimmunity, immune-mediated β-cell dysfunction and damage as part of their disease process. The progression to insulin dependence in LADA patients is believed more rapidly than classic type 2 diabetes patients who were negative for islet autoantibodies that have been proved with no progressive damage in beta cell [7]. However, the pathogenesis of LADA is still unclear, and the criteria for diagnosing the condition vary between studies. Therefore, the prevalence of LADA patients varies from 2.8 to 22.3 % in different published studies [8], and 8–10 % of patients diagnosed with T2D are in fact misdiagnosed LADA case on average. So, efforts on establishing a targeted treatment strategy and exploring the early detection for primary prevention have come under the spotlight.

It has been clearly identified that there is a strong genetic component affects diabetes. Genome-wide association studies (GWAS) have had considerable success in identifying genetic contributions to T1D and T2D. Unfortunately, LADA is not arousing our attention, and the genetic studies of LADA are sorely lacking. However, some newly articles reported that the single nucleotide polymorphism (SNP) of some genes that associated with T1D and T2D is also showed relevancy with LADA [9]. The protein tyrosine phosphatase N22 gene (PTPN22), which localized on chromosome 1p13 [10] and constituted by 21 exons [11], encodes a lymphoid-specific phosphatase known as LYP. It is a powerful inhibitor of T cell activation [12], which is fundamental for T cell proliferation and maturation [13]. Mutation of PTPN22 gene may potentiate T cell activation and induce autoimmune diseases. Several studies showed that splice variants of PTPN22 rs2476601 may associated with type 1 diabetes [14] and other autoimmune diseases [15, 16]. The cytotoxic T-lymphocyte antigen-4 (CTLA-4) is a co-stimulatory molecular, which is located on chromosome 2 (2q33) [17]. It encodes a glycoprotein receptor of the immunoglobulin (Ig) family expressed on the surface of activated T cells [18], act as an important negative regulator of T cell activation, playing a protective role in autoimmunity [19]. A single nucleotide polymorphism of CTLA-4 rs231775 has been identified as potential risk factors contributing to the development of T1D [17].

Latent autoimmune diabetes in adults (LADA) has been considered as a subgroup of type 1 diabetes in the World Health Organization (WHO) classification. A number of studies have assessed the association between the polymorphism of PTPN22 rs2476601/CTLA-4 rs231775 and LADA in different population [ 20–23]. However, the individual study may not have enough statistical power to detect a true association, and some of the results are inconsistent. Our aim is to estimating strength, accuracy and feature of the association of polymorphism in PTPN22 rs2476601 with LADA, and the relationship between CTLA-4 rs231775 and LADA, performing a meta-analysis of the available literature.

## Materials and methods

### Literature search

Systematic computerized searches (up to May 2013) without language limitation were performing by using PubMed, Web of knowledge and Chinese National Knowledge Infrastructure (CNKI). A combination of keywords was applied as follows: [(gene or allele or polymorphism) and (PTPN22 or protein tyrosine phosphatase N22) and (CTLA-4 or cytotoxic T-lymphocyte antigen-4)], [(PTPN 1858 or rs2476601)], [(CTLA-4 +49A/G or rs231775)] and [(LADA or latent autoimmune diabetes in adults)]. Only published articles were considered and set no restriction on the source of controls. We browsed the title and abstract of all related manuscripts, manually examined reference lists for additional citations and obtained the full text of all potentially relevant articles. If there were more than one articles published by the same content, we choose the most complete and recent study.

### Inclusion and exclusion criteria

Two reviewers (F.D. and W. K. L.) independently went through all titles and abstracts of the identified studies. Studies were selected if they met the following criteria: a case–control study that were written in English or Chinese; genotyped PTPN22 (PTPN22 1858 or rs2476601) or CTLA-4 (+49A/G or rs231775) polymorphisms and detailed data of each genotype; the outcome was latent autoimmune diabetes in adults (LADA); articles had to report the odds ratio and corresponding 95 % confidence interval or provided the sufficient information for estimation. Studies with insufficient data for pooling that with no frequencies of genotypes for each polymorphisms and outcomes were excluded.

### Data extraction

For quality control, information was extracted from the studies independently by two investigators (F.D. and H. W. P.). If lack of genotype information, we will try to contact the corresponding author in order to obtain required data. If they did not provide data, those studies were excluded from our review. General characteristics (e.g., the ethnic, genotyping method and the number of male) of included studies were extracted. Any disagreement was resolved by consensus.

### Risk of bias assessment

The quality of studies was also independently assessed by the same reviewer (F.D. and G. Y.) based on a risk of bias score for genetic association. This was modified on the basis of both traditional epidemiologic considerations and genetic issues, which were developed by Thakkinstian et al. [29]. The score was divided into five domains, including information bias, confounding bias, selective reporting of outcomes, population stratification and assessment of Hardy–Weinberg equilibrium (HWE) in the control group. Each item was classified with regard to “yes” or “no” or “unclear,” which represent low risk, high risk and insufficient information, respectively. Disagreement between the two reviewers was solved by a senior reviewer (C. X. J.).

### Statistical analysis

We used the Comprehensive Meta-Analysis software (ver2.0) for all statistical analyses. The Hardy–Weinberg equilibrium (HWE) was examined in control groups by Fisher’s exact test. If the study was found not to be in HWE with *P* value less than 0.05, it was considered to be disequilibrium. We performed both per-allele and per-genotype approaches to estimate the strength of association between the polymorphism of genes and LADA risks.

#### Per-allele analysis

Suppose that D and d are risk and non-risk alleles, and DD, Dd and dd are minor homozygous, heterozygous and common homozygous genotype, respectively, for each polymorphism. The risk allele frequency in each group was estimated for each study by reported genotype data, and overall prevalence along with 95 % confidence intervals was estimated for each SNP. The Z-test was used to determine the statistical significance of the pooled OR, and its *P* value was used to determine whether the overall gene effect was significant (*α* = 0.05). Heterogeneity of odds ratios across studies was calculated by a Q test, and the degree of heterogeneity was quantified by *I* ^2^ test [30]. If the inspection result shows *P* > 0.10, a fixed-effect model was selected to pool the data, which can be considered as the evidence of homogeneity between studies. Otherwise, a random-effect model was used. In addition to this, the degree of heterogeneity was quantified using *I* ^2^ (*I* ^2^ < 25 %, no heterogeneity; 25 % < *I* ^2^ < 50 %, moderate heterogeneity; 50 % < *I* ^2^ < 75 %, large heterogeneity; and *I* ^2^ > 75 % extreme heterogeneity) [31]. We choose a random-effect model if *I* ^2^ was greater than 50 % [32]. If there is high heterogeneity exists, a set of subgroup meta-analysis were considering exploring the heterogeneity of current sources by ethnic group. The population-attributable risk (PAR) for risk allele was calculated based on results from discrete-time model [33, 34]. If the main effect of the genotype was statistically significant and with the appropriate effect model selection, further comparisons of OR1 and OR2 were explored.

#### Per-genotype analysis

We perform the model-free approach to estimate the genotype effect [35], two odds ratios: DD versus dd (OR1) and Dd versus dd (OR2) were estimated for each study. The model of genetic effect, measured by the parameter lambda (*λ*), which is defined as the ratio of logOR2 to logOR1, was then estimated using the model-free Bayesian approach. This parameter ranges from 0 to 1, which represents the heterozygote effect as a proportion of the homozygote variant effect and captures information about the genetic mode of action as follows: If *λ* = 0, a recessive (DD vs. Dd +dd) model is suggested; if *λ* = 1, a dominant model (DD + Dd vs. dd) is suggested; and if *λ* = 0.5, a codominant model (DD vs. dd; Dd vs.dd) is suggested. If *λ* > 1 or *λ* < 0, then a homozygous or heterosis model is likely, although this is rare. The two log odds ratios are modeled as either fixed or random effects, as described in the second statistical analysis enumerated above. Once the best genetic model is identified, this model is used to collapse the three genotypes into two groups and to pool the results again. For lambda, WinBugs 1.4.2 was used with vague prior to distributions for estimation of parameters (i.e., lambda and odds ratio). The models were run with a burn-in of 1,000 iterations, followed by 10,000 iterations for parameter estimates.

Publication bias was assessed using the cumulative forest plot and Egger’s regression intercept [36]. Cumulative forest plot can reflect the dynamic change trend of the research results and the potential impact of small samples on estimate effect size [37]. We did a sensitive analysis to estimate the stability of the meta-analysis with two statistical methods. We first omitted one study and observed the influence of the remaining results to the overall OR, and fail-safe number was also used to estimate the stability of the results.

## Result

### Characteristics of the studies

Thirteen relevant articles were identified after the primary literature search about PTPN22; seven articles were excluded after screening abstracts and full texts. Among these articles, some are described the irrelevant content to the LADA topic and others are lack of the detailed data we required. Finally, only six articles were left. There were 22 studies conform to the standard after the preliminary search about CTLA-4 gene. After extraction, a total of six case–control studies were selected according to the search criteria for LADA related to the polymorphism of CTLA-4. HWE was calculated for control groups in all articles; we found that one study was showed disequilibrium (Liu [25], *P* < 0.001).

### Risk of bias assessment

As shown in the “Appendix,” the criteria for evaluating the quality of cases and controls were clearly described for all included studies. This work was conducted by two reviewers, and the disagreement was solved by consensus and discussion. The risk of bias was highest in the quality control for genotyping (unclear in 7 out of 10 studies, or 70 %), followed by not assessing HWE (4/10, 40 %) and confounding bias (3/10, 30 %).

### Meta-analysis of PTPN22 rs2476601

**Fig. 1:**
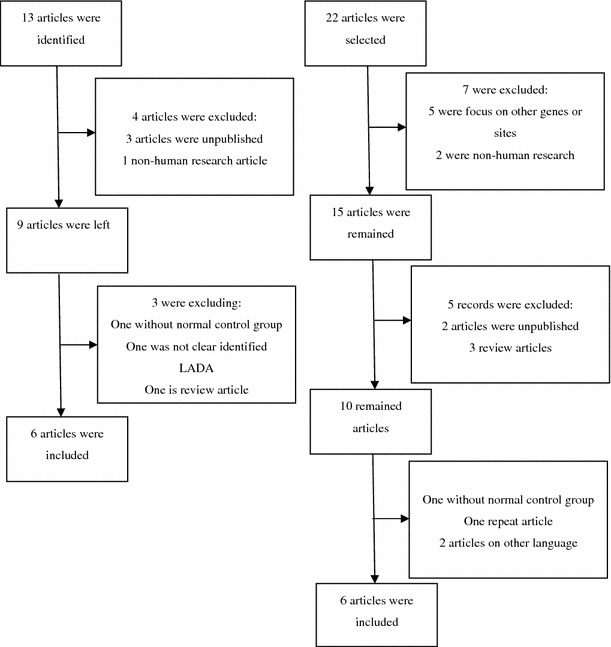
Flowchart for identify relevant studies for PTPN22 gene, CTLA-4 gene polymorphisms with LADA

**Fig. 2:**
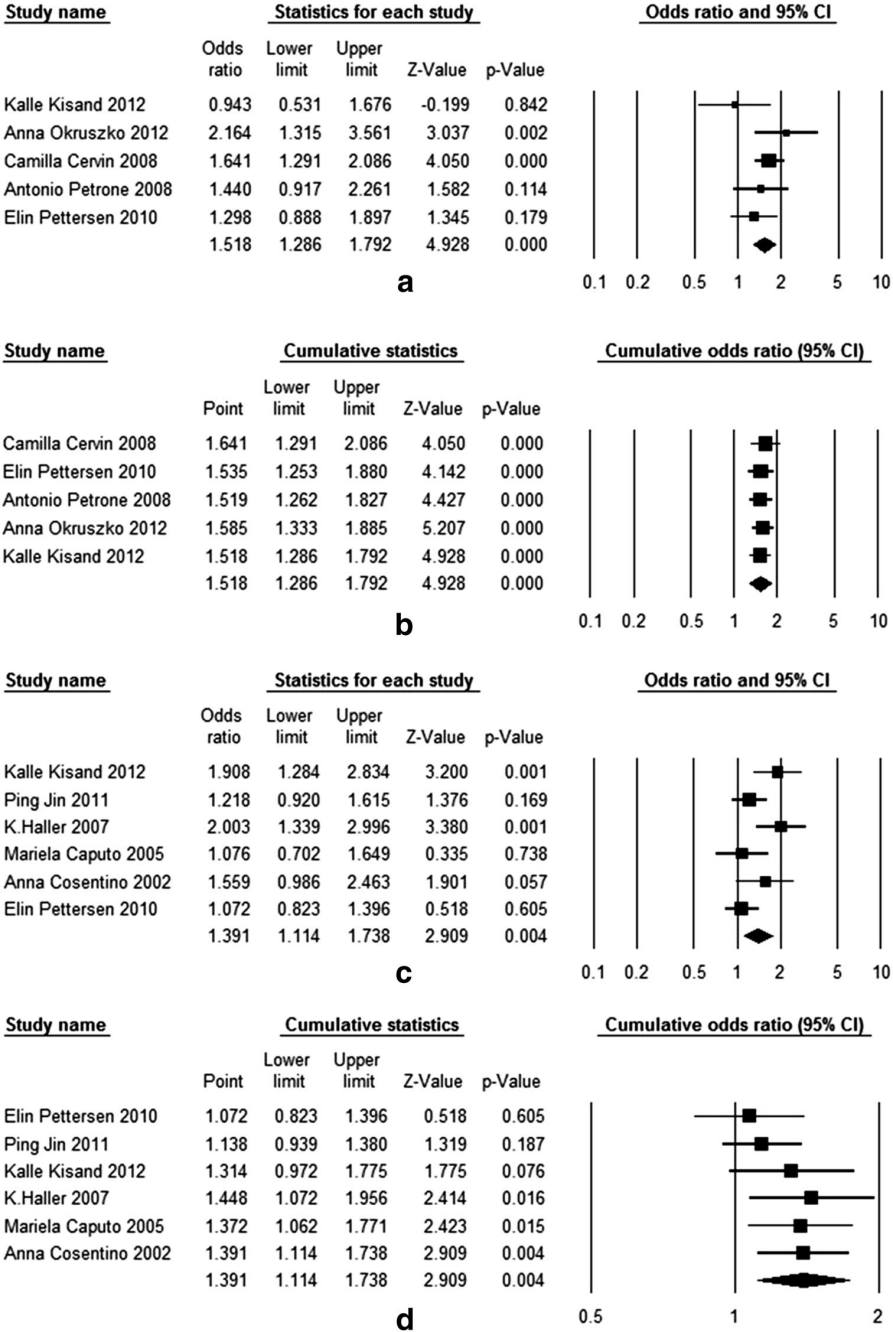
Forest plot and cumulative forest plot of PTPN22 and CTLA-4 genes with LADA. **a** Forest plot of the association between PTPN22 rs2476601 polymorphism and LADA risk (T vs. C), which was estimated by fixed-effect model. **b** Cumulative forest plot of PTPN22 gene (T vs. C). **c**. Forest plot of the association between CTLA-4 rs231775 polymorphism and LADA risk (G vs. A), which was calculated by random-effect model. **d**. Cumulative forest plot analysis of CTLA-4 gene (G vs. A). The size of each square is the proportion of percent weight of each study that contributed in the pooled odds ratio. The pooled odds ratios are indicated by the *diamond*. *Horizontal bars* represent the 95 % CI

There were six case–control studies described the association between PTPN22 rs2476601 polymorphism and LADA, which included 1,088 cases and 4,079 controls (Tables 1, 2). All except one study [25] did not observe HWE, and thus, this study was not included in further pooling. Results for these studies are summarized in Table 3. The pooled frequency of minor T allele was 16.9 % (95 % CI 9.7–24.0) in LADA group (*I* ^2^ = 92) and 9.9 % (95 % CI 7.6–12.7) in non-LADA group (*I* ^2^ = 91), which were both estimated by random model. The odds ratios (T vs. C) were not heterogeneous (*χ* ^*2*^ = 5.69, *P* = 0.34, *I* ^2^ = 12.14), with a pooled odds ratio of 1.52 (95 % CI 1.29–1.79). The overall gene effect estimated by fixed-effect model was significant (*P* < 0.001). This suggested that individuals carrying the minor T allele had 52 % increased risk of developing LADA than those carrying the major C allele (Table 4). Cumulative meta-analysis was performed for pooled odds ratio, which was used to estimate the publication bias (Fig. 1). From the shape of cumulative forest plot, we know that the point estimate of effect size is very stable transformation, after the process of article size in accordance with the order of accuracy gradually incorporated into the calculation model, which implied that there is no publication bias (seen in Fig. 2). The Egger test did not suggest any evidence of publication bias (SE = 1.79, *P* = 0.54). The sensitive analysis was performed by omitting one study at a time, which the method was used to make sure that no individual study was entirely responsible for the combined results. From the Table 5, we could found that none of the individual studies affect the final conclusion obviously about the gene. Classic fail-safe N value of PTPN22 rs2476601 was 21 (*P* = 0.00004, *Z* = 4.12) when α was set to 0.05, which suggest that 21 unpublished negative studies would have to be included to convert the combined *P* value to a nonsignificant value. The above results show that our results were statistically reliable.

**Table 1:** Characteristics of the selected study

Author	Year	Gene locus	Ethnic	Method	Male/number		Mean age		BMI			HWE	Confirmed standard	
					Case	Control	Case	Control		Case	Control		Case	Control (sources)
Kisand [20]	2012	rs2476601/rs231775	Caucasian	RFLP/TaqMan	25/65	91/229	54.5	45.9	NA		NA	Yes	Initially diagnosis of T2DM but with positive antibody (ICA/IA2A/GADA), no insulin treatment for at least 6 months	Healthy blood donors and hospitalized with no diabetes
Okruszko [24]	2012	rs2476601	Caucasian	PCR	42/80	NA/151	45.4	NA	NA		NA	Yes	WHO criterion	Medical staff and medical students with no family history or autoimmune disease
Liu [25]	2012	rs2476601	Asian	PCR	129/229	72/210	51.8	47.2	22.0 ± 4.2		22.4 ± 2.9	No	Age at onset > 35 year, positive at least one positive antibody (GAD-Ab/IA2-Ab), excluded other types of diabetes	Healthy volunteers with normal in OGTT test and no history of autoimmune disease or negative in antibody test
Cervin [9]	2008	rs2476601	Caucasian (Swedish)	MALDI-TOF–MS	73/164	553/1000	52.4	70.1	25.9 ± 5.6		27.6 ± 4.4	Yes	Age at onset > 35 year, GAD antibody positive	Without family history of diabetes or treatment of hypertension
			Caucasian (Finnish)	MALDI- TOF–MS	83/113	83/113	54.6	53.7	26.8 ± 5.0		25.9 ± 3.7	Yes		
Petrone [21]	2008	rs2476601	Caucasian	PCR	131/250	278/545	50.3	30.0	NA		21.8 ± 2.2	Yes	Initial diagnosis of T2DM, and with positive GADAs antibody, no insulin requirement and no ketosis disease duration between 6 months to 5 years	Normoglycemic subjects with no family history of autoimmune disease
Pettersen [22]	2010	rs2476601/rs231775	Caucasian	TaqMan	68/126	740/1503	NA	NA	NA		NA	Yes	Anti-GAD positive, no insulin treatment within 12 months	A questionnaire with answer “no” of the question: having diabetes?
Jin [23]	2011	rs231775	Asian	PCR	79/135	297/476	49.6	48.2	NA		NA	Yes	Immunology of Diabetes Society criterion	Non-diabetic individuals with no family history and no autoimmune disease
Haller [26]	2007	rs231775	Caucasian	RFLP	24/61	101/252	64.6	45.5	NA		NA	Yes	T2DM patients with at least on positive antibody (ICA/IA-2/GAD 65)	Younger: the blood donation, older: hospitalized for various reasons, without T1DM and T2DM
Caputo [27]	2005	rs231775	Caucasian	PCR	NA/63	NA/168	51.4	NA	NA		NA	Yes	WHO criterion	Healthy person with no family history and normally fasting blood glucose level
Cosentino [28]	2002	rs231775	Caucasian	PCR	22/80	42/85	51	48	NA		NA	Yes	Initially classified as T2DM, and with islet antibodies	Employees of the university with no family history of diabetes

*BMI* body mass index, *RFLP* restriction fragment length polymorphism analysis, *MALDI-TOF–MS* matrix-assisted laser desorption/ionization time of flight mass spectrometry, *PCR* polymerase chain reaction, *NA* not available

**Table 2:** The risk of bias assessment

Author	Ascertainment of LADA	Ascertainment of control	Quality control for genotyping	Population stratification	Confounding bias	Selective outcome report	HWE
Kisand [20]	Yes	Yes	Yes	Yes	No	Yes	Yes
Okruszko [24]	Yes	Yes	Unclear	Yes	No	Yes	No
Liu [25]	Yes	Yes	Unclear	Yes	Yes	Yes	Yes
Cervin [9]	Yes	Yes	Yes	Yes	Yes	Yes	Yes
Petrone [21]	Yes	Yes	Unclear	Yes	Yes	Yes	Yes
Pettersen [22]	Yes	Yes	Yes	Yes	Yes	Yes	Yes
Jin [23]	Yes	Yes	Unclear	Yes	Yes	Yes	Yes
Haller [26]	Yes	Yes	Unclear	Yes	Yes	Yes	No
Caputo [27]	Yes	Yes	Unclear	Yes	No	Yes	No
Cosentino [28]	Yes	Yes	Unclear	Yes	Yes	Yes	No

**Table 3:** Genotype frequencies for PTPN22 rs2476601 and genotype effects of studies included in the meta-analysis

Author	Total number		Case genotype			Control genotype			T allele prevalence	T versus C			TT versus CC			CT versus CC			HWE
	Case	Control	TT	CT	CC	TT	CT	CC		OR	95 % CI		OR	95 % CI		OR	95 % CI		
Kalle Kisand	65	229	1	15	49	6	51	172	0.138	0.943	0.531	1.676	0.585	0.069	4.976	1.032	0.535	1.992	*P* = 0.40
Anna Okruszko	80	151	9	20	51	1	36	114	0.126	2.164	1.315	3.561	20.118	2.483	163.008	1.242	0.656	2.352	*P* = 0.47
Camilla Cervin	341	1,453	7	92	242	18	257	1,178	0.101	1.641	1.291	2.086	1.893	0.782	4.582	1.743	1.323	2.295	*P* = 0.38
Antonio Petrone	250	545	1	31	218	2	47	496	0.047	1.440	0.917	2.261	1.138	0.103	12.612	1.501	0.928	2.427	*P* = 0.33
Elin Pettersen	123	1,491	1	32	90	18	292	1,181	0.110	1.298	0.888	1.897	0.729	0.096	5.523	1.438	0.942	2.196	*P* = 0.99
Overall odds ratio										1.518	1.286	1.792	1.861	0.940	3.684	1.523	1.260	1.840	

*HWE* Hardy–Weinberg equilibrium

**Table 4:** Genotype frequencies for CTLA-4 rs231775 and genotype effects of studies included in the meta-analysis

Author	Total number		Case genotype			Control genotype			G allele prevalence	G versus A			GG versus AA			GA versus AA			HWE
	Case	Control	GG	AG	AA	GG	AG	AA		OR	95 % CI		OR	95 % CI		OR	95 % CI		
Kalle Kisand	65	229	21	35	9	37	124	68	0.432	1.908	1.284	2.834	4.288	1.783	10.313	2.133	0.968	4.699	*P* = 0.14
Ping Jin	135	476	51	73	11	167	239	70	0.602	1.218	0.920	1.615	1.943	0.957	3.948	1.944	0.977	3.866	*P* = 0.34
K.Haller	61	252	20	33	8	40	135	77	0.427	2.003	1.339	2.996	4.812	1.948	11.891	2.353	1.035	5.350	*P* = 0.16
Mariela Caputo	63	168	6	35	22	21	76	71	0.351	1.076	0.702	1.649	0.922	0.331	2.572	1.486	0.797	2.773	*P* = 0.99
Anna Cosentino	80	85	4	55	21	5	40	40	0.294	1.559	0.986	2.463	1.524	0.369	6.285	2.619	1.344	5.103	*P* = 0.30
Elin Pettersen	124	1,477	48	52	24	499	715	263	0.580	1.072	0.823	1.396	1.054	0.632	1.759	0.797	0.482	1.319	*P* = 0.83
Overall odds ratio										1.391	1.114	1.738	1.961	1.098	3.502	1.682	1.119	2.527	

*HWE* Hardy–Weinberg equilibrium

**Table 5:** The result of sensitive analysis

Gene	Excluded study	Pooled OR	95 % CI	*P*	*I* ^2^ (%)	*P* value for *I* ^2^
PTPN22 rs2476601	Okruszko [24]	1.453	1.218–1.732	<0.001	0.00	0.477
	Petrone [21]	1.531	1.281–1.829	<0.001	28.94	0.229
	Cervin [9]	1.414	1.124–1.778	0.003	18.58	0.296
	Pettersen [22]	1.575	1.310–1.894	<0.001	18.05	0.300
	Kisand [20]	1.585	1.333–1.885	<0.001	0.00	0.588
CTLA-4 rs231775	Cosentino [28]	1.314	1.132–1.526	0.001	63.688	0.026
	Pettersen [22]	1.460	1.235–1.728	<0.001	48.397	0.101
	Haller [26]	1.262	1.084–1.468	0.003	43.419	0.132
	Kisand [20]	1.268	1.089–1.476	0.002	49.531	0.094
	Caputo [27]	1.372	1.181–1.595	<0.001	61.487	0.034
	Jin [23]	1.379	1.170–1.624	<0.001	63.459	0.027

Genotype frequency and estimated OR for each study were shown in Table 3. The OR1 for TT versus CC was moderate heterogeneity (*χ* ^2^ = 7.08, *P* = 0.13, *I* ^2^ = 43.51), whereas the OR2 for CT versus CC was homogenous (*χ* ^2^ = 2.86, *P* = 0.72, *I* ^2^ = 0.00). They both calculated by fixed-effect model. The pooled OR1 and OR2 were 1.86 (95 % CI 0.94–3.68) and 1.52 (95 % CI 1.26–1.84), respectively, which suggested that individuals with TT and CT genotypes had 86 and 52 % higher risk of LADA than those carrying CC genotype. The *λ* = 0.49 (95 % CI 0.07–0.96) which suggested that a codominant effect was most likely, although one genotype effect did not reach statistical significant.

### Meta-analysis of CTLA-4 rs231775

The associations between CTLA-4 rs231775 and LADA were investigated in six case–control studies, with 528 cases and 2,687 controls. The pooled frequency of minor G allele in LADA group was 49.2 % (95 % CI 35.2–63.3), along with high heterogeneity (*I* ^2^ = 95), and in non-LADA group was 44.9 % (95 % CI 36.3–53.9), which estimated by random model (*I* ^2^ = 97). The pooled OR was calculated by random-effect model due to the high heterogeneity (*χ* ^2^ = 11.50, *P* = 0.04, *I* ^2^ = 56.51). The odds ratio of G versus A is 1.39 (95 % CI 1.11–1.74) with statistical significance (*P* = 0.004), which indicated that individuals carrying G allele had 39 % increased risk of developing LADA than those carrying A allele. In cumulative plot, the summary OR was a bit different in the first two studies, whereas not much changed in a smooth curve with the increase of the literature. Egger’s regression test also suggest no publication bias (SE = 2.50, *P* = 0.18). In the sensitive analysis, after each removed a piece of literature have not seen a big difference in the OR values have changed. Classic fail-safe N value of CTLA-4 rs231775 is 24 (*P* = 0.00001, *Z* = 4.37) when α was set to 0.05, which suggest that 24 unpublished negative studies would have to be included to convert the combined *P* value to a nonsignificant value. This shows that our results are stable enough. When studies were divided according to the ethnic group, the result showed that there is a significant association in Caucasian. The pooled odds ratio is 1.45 (95 % CI 1.09–1.92), with a significantly statistical gene effect (*P* = 0.01) but a highly heterogeneity (*I* ^2^ = 63.46, *P* = 0.03). There is only one Chinese study that belongs to Asian population with no statistical significance (OR 1.22, 95 % CI 0.92–1.62, *P* = 0.17).

In heterogeneity test, there is a moderate heterogeneity across OR1 (*χ* ^2^ = 14.19, *P* = 0.01, *I* ^2^ = 64.77) and OR2 (*χ* ^2^ = 11.12, *P* = 0.05, *I* ^2^ = 55.04). The summary odds ratios for the GG and AG genotype were estimated by random model, they are 1.96 (95 % CI 1.10–3.50) and 1.68 (95 % CI 1.12–2.53), respectively. These point estimates can be interpreted as that person with the GG and GA genotypes had 96 and 68 % higher risks of developing LADA than persons with the AA genotype. The estimated *λ* = 0.63 (95 % CI 0.15–0.98) which suggested that a codominant effect was most likely.

## Discussion

We performed a systematic review and meta-analysis to determine the effects of two gene polymorphisms (PTPN22 rs2476601 and CTLA-4 rs231775) on the LADA. The analyses included pooling data from five and six studies with a total sample size of 4,728 and 3,215 subjects. We were able to identify PTPN22 rs2476601 and CTLA-4 rs231775 polymorphisms as genetic markers that might increase the risk of LADA. Individuals who carried minor allele T in rs2476601 had 52 % increased risk of developing LADA relative to those carrying C allele, while individuals carrying the risk allele G in rs231775 may lead to an increasing risk of having LADA by 39 % compared with allele A. The results suggest association in Caucasians, that is, carriage of G in the CTLA-4 rs231775 increases 45 % relative to carriage of A allele. However, Asian populations showed an unrelated result. This difference may be due to the different genetic backgrounds and limited article.

The minor T risk allele of the PTPN22 rs2476601 polymorphism investigated is quite rare in non-LADA group, with frequency of 9.9 %. However, it is high in the LADA group, with frequency of 16.9 %. The PAR for the minor T was 4.88 %, which suggested that PTPN22 rs2476601 polymorphism probably serves as a marker for an absolute lowering of the risk of all LADA in Caucasians by 4.88 % when individuals do not carry T allele. The risk G allele in CTLA-4 rs231775 is common, with similar frequencies of 44.9 and 49.2 % in non-LADA group and LADA group, which might indicate an important effect at a population level. The PAR of CTLA-4 G allele was 14.93 %, which may provide a useful clinical estimation that might contribute an absolute lowering of the risk of all LADA by 14.93 % when individuals do not carry this allele.

Genotypic effects were also estimated for PTPN22 rs2476601 and CTLA-4 rs231775. For PTPN22 rs2476601, the estimated OR1 for TT versus CC and OR2 for CT versus CC were 1.86 and 1.52 in Caucasian, respectively, and estimated lambda was 0.49, suggesting a codominant mode of gene effect. However, the 95 % confident interval of lambda laid from 0.07 to 0.96, which suggested that the genetic mode could be recessive dominant and codominant. This pooling was based on small number of included studies, and thus, uncertainty of gene effects was still present. For CTLA-4 rs231775, the genotype effects of GG and GA versus AA were 0.96 and 0.68, respectively. The point estimated lambda was 0.63, suggesting a codominant mode of gene effect. The 95 % confident interval of lambda laid from 0.15 to 0.98, which suggested that the genetic mode could be recessive dominant and codominant.

Previous studies have shown that the SNPs of PTPN22 and CTLA-4 are associated with T1DM [38–43] and other autoimmune diseases [44–47], which have some meta-analysis to support it [48–51].Our research showed statistical evidence that the polymorphism of genes PTPN22 rs2476601 and CTLA-4 rs231775 is associated with LADA on the basis of population study, which could provide some clues on the research of fundamental to diabetes biology and uncover the major genetic factors involved in the pathogenesis of LADA.

LADA is a common subgroup of diabetes accounting for about 7 % of all diabetic patients in Europe (http://andis.ludc.med.lu.se). Multiple islet auto-antigens and autoantibodies could be detected before the development of autoimmune diabetes [52, 53], such as cytoplasmic islet cell autoantibodies (ICA) and glutamic acid decarboxylase autoantibody (GADA), which have been recognized as the most effective immune marker for LADA diagnosis [54, 55]. Huang Gan et al. also reported that combination testing of IAA with GADA and IA-2A could improve LADA diagnose rate by 2.39 % than GADA and IA-2A, which increased the evidence that autoimmunity to insulin may be central to disease pathogenesis [56]. It though should be noted that to date, no GWAS has been performed on LADA patients. Even though some newly articles reported that the single nucleotide polymorphism (SNP) of some genes that associated with T1D and T2D is also showed relevancy with LADA [9], the possible reason for inconsistence could be due to the diagnostic criteria for LADA or distinct interactions of genes and environment.

Heterogeneity is a potential factor affecting pooled results [57], which can be divided into genetic heterogeneity of effect and the genetic heterogeneity of the model. In our meta-analysis, a small heterogeneity was proved in the analysis of PTPN22 rs2476601 polymorphism with LADA in per-allele analysis; all studies included Caucasians, which may produce better consolidation effect. Beyond that, we excluded one study [25] which is out of Hardy–Weinberg equilibrium in the control group when we do the pooled odds ratio in order to make the results more precise. However, in the research of CTLA-4 rs231775 polymorphism with LADA, the result was suffered moderate heterogeneity influenced. We do a subgroup analysis according to ethnic population to explore the source of heterogeneity, and highly heterogeneity was observed in Caucasian groups. When we exclude the article by Elin [22] and Haller [26] during the sensitive analysis, the *I* ^2^ reduced and we conjecture that these two articles may increases the overall heterogeneity when we do the pooling. This analysis implies that different genetic backgrounds and small study sample size may be the source of heterogeneity. Analyses based on specific genetic models can produce misleading estimates of the odds ratios when an inappropriate model is assumed. The pooled genetic association was calculated by a genetic model-free approach, which does not assume that the underlying genetic model is known in advance but still makes use of the information available on all genotypes. We avoided multiple comparisons, which would lead to overly strong misjudge assumptions about the genetic model or of inefficient estimates, and offer a single method that could have been used in all of these examples giving a consistent presentation and to reduce heterogeneity.

There are still some limitations in our article. Firstly, we just conducted in English and Chinese literature retrieval, which may result in missing some related articles written by other languages. Secondly, the sources of control are not clearly and uniform that might lead to not enough estimation. Another potential disadvantage is that all the included studies were case–control study, which might overestimate the genetic association. To avoid such bias, the best way is to establish the population-based nested case–control study, although it is hard to implement. The last limitation is small sample size. There were only six studies included in two gene polymorphisms, which may lead to not powerful enough estimation. The small sample size study may have a low power and affecting the results in the process of pooled odds ratio. So a more precise association needs to be explored further with sufficient data. Thus, our results should be interpreted with caution until further verification of sequencing approaches plus larger and larger meta-analysis.

In conclusion, our meta-analysis suggests that both of the PTPN22 rs2476601 and the CTLA-4 rs231775 polymorphisms contribute to susceptibility to LADA. Future large, well-designed studies are warranted to examine the impact of PTPN22 and CTLA-4 on LADA risk. What’s more, a better understanding of the genetic basis is needed to more accurately place this disorder in the spectrum of diabetes phenotypes, which further research on genome-wide genotyped datasets, and more detailed genetic studies of LADA could help unravel the genetic etiology of LADA. In addition, a comprehensive interaction on gene–gene and gene–environment should also be evaluated in future analysis.

### Electronic supplementary material

Below is the link to the electronic supplementary material.

Supplementary material 1 (DOCX 88 kb)

## Appendix

Table 6.

**Table 6:** Risk of bias assessment for genetic association studies of LADA of studies included in the meta-analysis

Domain and item	Low risk of bias
*Information bias*	
Ascertainment of LADA	
Clearly described objective criteria of diagnosis of LADA	Yes
Not clearly described	No
Did not mention	Unclear
Ascertainment of controls	
Controls were non-LADA and without family history	Yes
Mentioned the sources of controls	Yes
Not described	No
Ascertainment of genotyping examination	
Genotyping done under “blind” conditions of case specimens and control specimens	Yes
Genotyping of cases and controls was performed together	Yes
Genotyping error rate <5 %	Yes
Quality control procedure (e.g., reanalysis of random specimens, by using different genotyping methods for analysis, analysis if replicate sample)	Yes
Unblind	No
Genotyping error rate >5 %	No
Did not mention what was done	Unclear
*Confounding bias*	
Population stratification	
No difference in ethnic origin between cases and controls	Yes
Use of controls who were not related to cases with clearly identification	Yes
Use of some controls who came from the same family	No
No report of what was done	Unclear
Other confounding bias	
Controls for confounding variables (e.g., age, gender, or BMI) in analysis	Yes
Not controlled for confounding variables	No
Not mentioned	Unclear
Selective reporting (for replication studies)	
Reported results of all polymorphisms mentioned in the objectives, no significant or not	Yes
Reported results of only significant polymorphisms	No
HWE	
HWE in the control group	Yes
HWD in the control group	No
HWE not checked or mentioned	No

## Abbreviations

LADA: Latent autoimmune diabetes in adults
GWAS: Genome-wide association study
SNP: Single nucleotide polymorphism
BMI: Body mass index
HWE: Hardy–Weinberg equilibrium
PAR: Population-attributable risk
