# Supplementary material for: The association of PTPN22 rs2476601 polymorphism and CTLA-4 rs231775 polymorphism with LADA risks: a systematic review and meta-analysis
Source: Acta Diabetol. 2014 Jul 9;51(5):691–703. doi: 10.1007/s00592-014-0613-z (PMC4176954; doi:10.1007/s00592-014-0613-z)
Supplement: Supplementary file 1 — Supplementary material 1 (DOCX 88 kb) [file 592_2014_613_MOESM1_ESM.docx]

**Supplementary**

**
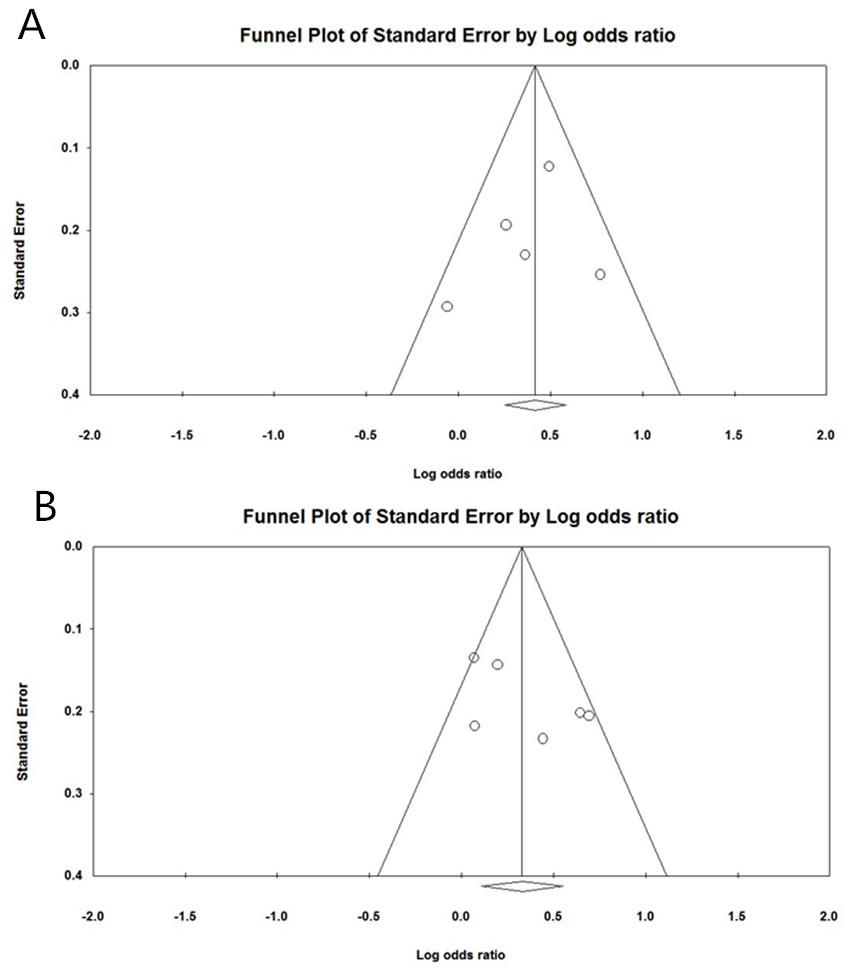
**

**Supplementary Figure 1.** The funnel plots analysis to detect publication bias

A. Funnel plots of PTPN22 rs2476601 for T vs. C; B. Funnel plots of CTLA-4 rs231775 for G vs. A. Each circle represents an independent study for the indicated association.
